# Rethinking digital and AI inclusion: participatory and intersectionality-informed methods for disability and migrant justice

**DOI:** 10.3389/fsoc.2025.1593330

**Published:** 2025-07-21

**Authors:** Karen Soldatic, Mikyung Lee, Eunice Tunggal, Ashley Liao, Liam Magee

**Affiliations:** ^1^Canada Excellence Research Chair—Health Equity and Community Wellbeing, School of Disability Studies, Toronto Metropolitan University, Toronto, ON, Canada; ^2^Institute of Culture and Society, Western Sydney University, Penrith, NSW, Australia; ^3^College of Education, University of Illinois Urbana-Champaign, Champaign, IL, United States

**Keywords:** disability, migration, digital, inclusion, engagement, accessibility, AI, technology

## Abstract

Everyday consumer technologies are increasingly integral to autonomy, mobility, and social participation among people with disabilities and migrants from culturally and linguistically diverse (CaLD) backgrounds. However, these technologies often remain inaccessible and exclusionary at the intersection of these identities. This study examined how CaLD migrants with disabilities engage with everyday consumer technologies using participatory and intersectionality-informed approaches. This article focuses on Stage Two of the Autonomy, Diversity & Disability: Everyday Practices of Technology project, funded by the Australian Research Council industry partnership grant (LP: 190900099), which involved individual interviews, creative workshops, guided discussions, post-workshop reflections, and the co-creation of AI-generated e-books. Drawing on three case studies, the analysis identified three key findings: (1) participants experienced a disproportionate burden in navigating digital accessibility and advocating for their needs; (2) generative AI perpetuated biases and misrepresentations of intersecting identities; and (3) participants actively used everyday consumer technologies to foster agency, learning, caregiving, and cultural connection. Through sustained participatory engagement, the researchers identified methodological parameters to inform future disability-inclusive, participatory, and intersectionality-informed research.

## 1 Introduction

Everyday consumer technologies, such as smartphones, messaging apps, and GPS navigation, have become deeply embedded into everyday life, shaping how individuals connect, navigate, and access essential services across digital and physical spaces. For individuals with disabilities, these technologies support decision-making, mobility, and communication, which collectively foster greater autonomy (Al Zidjaly, 2015; Steel, 2019), demonstrating their transformative potential in everyday life and fostering empowered, independent living (Ellis, 2016; Goggin, 2017; Ellis and Kent, 2016, 2010). Similarly, for culturally and linguistically diverse (CaLD) migrants, such technologies play a critical role in resettlement, social participation, and transnational connections (SSI, 2018; Caluya et al., 2018).

In Australia, CaLD migrants constitute one of the fastest-growing population groups, particularly in metropolitan and peri-urban regions such as Sydney and Melbourne, where established migrant communities provide critical social, economic, and cultural support (Australian Bureau of Statistics, 2021; SSI, 2018). Sydney, in particular, has seen an increase in concentrated ethnic communities, where everyday consumer technologies serve as a crucial link to public and private services, intra-community communication, and socio-economic participation (Pasquale, 2015; Caluya et al., 2018). However, despite these demographic shifts, Australia's digital infrastructures and mainstream everyday consumer technologies remain predominantly designed for an Anglo-centric user base, failing to adequately address the cultural, linguistic and accessibility needs of CaLD migrants.

This exclusionary nature is further compounded by algorithmic biases embedded in artificial intelligence (AI) systems, which are often trained on datasets that perpetuate linguistic and ableist biases (Pasquale, 2015; Noble, 2018). These biases situate everyday consumer technologies within socio-technical systems that reinforce existing power hierarchies rather than universally accessible tools they are intended to be (Heeks, 2022; Goggin, 2017). Of particular concern is the exclusion of CaLD migrants with disabilities—especially those who acquire disability before the age of 65. They remain one of the most under-serviced and under-resourced user groups in Australia (Soldatic et al., 2014; SSI, 2018) which further exacerbates their exclusion from essential digital and public services [Women With Disabilities Australia (WWDA), Harmony Alliance and National Ethnic Disability Alliance (NEDA), 2023].

While a growing body of research has examined disability and CaLD migration in relation to technology engagement (Al Zidjaly, 2015; Watermeyer and Goggin, 2019; Whitehead et al., 2023), these studies have largely examined disability and CaLD migration as distinct areas of inquiry, with limited attention given to their intersection (Swartz and Marchetti-Mercer, 2019). Recent pilot research conducted in Australia (Soldatic et al., 2020) further underscore these gaps, revealing that service providers and users alike report that everyday consumer technologies are inadequately adapted to the needs of CaLD migrants with disabilities, making them inaccessible, unaffordable, and stigmatizing. Users emphasized that technological developments aimed at improving access to digital and public services often addressed either their CaLD migrant identity or their disability but rarely considered the intersection of both. Consequently, individuals at this intersection face substantial accessibility and usability barriers, ultimately limiting the transformative potential of everyday consumer technologies (Parette and Scherer, 2004).

Addressing these structural limitations, an intersectional lens provides a critical framework for understanding how multiple intersecting systems of power shape the technology experiences of CaLD migrants with disabilities. First articulated by the Combahee River Collective (1977) and later formalized as a theoretical framework by Crenshaw (1991), intersectionality critiques single-axis approaches to oppression and emphasizes that overlapping systems of power interact to create distinct and compounded forms of exclusion. This framework has since been widely adopted across disciplines, including disability studies, where scholars emphasized the necessity of examining multiple dimensions of systemic oppression to understand the lived realities of individuals with disabilities (Wolbring and Nasir, 2024). However, disability-digital research remains largely situated within a Western, Anglo-centric paradigm that overlooks the intersections of disability and CaLD identity, thereby perpetuating the exclusion of CaLD migrants with disabilities from digital infrastructures and mainstream technologies.

In response to these challenges, the Autonomy, Diversity & Disability: Everyday Practices of Technology (ADDEPT) project, funded by the Australian Research Council industry partnership grant (LP: 190900099), was established to explore the intersections of disability and CaLD migrant identity in relation to technology engagement through an intersectional lens. Conducted between 2020 and 2023, the project explored how CaLD migrants with disabilities navigate, engage with, and adapt everyday consumer technologies in their daily lives. Utilizing participatory, co-creation approach, the project engaged CaLD migrants with disabilities, community leaders, and service providers to critically examine accessibility barriers, digital participation, and systemic exclusion that shape their technological interactions. The ADDEPT project was structured into five phases (Table 1), each designed to investigate different aspects of technology engagement among CaLD migrants with disabilities.

**Table 1:** ADDEPT project outline.

	**Stage one**	**Stage two**
Phases	1, 2, and 5	3 and 4
Data collection methods	Focus groups, interviews, and one-day forum	Interactive workshops, self-reflective storytelling, and AI-assisted media production
Participants	CaLD migrants with disabilities, Community leaders, disability advocates, peer support advocates, representatives from service providers and the technology industry	CaLD migrants with disabilities (subset of Stage One participants)

Stage one (Phases 1, 2, and 5) focused on identifying digital barriers, gathering empirical insights, and translating findings into policy and practice recommendations through participatory workshops, focus groups, and interviews. Stage Two (Phases 3 & 4) adopted an innovative and experiential approach by integrating interactive workshops, self-reflective storytelling, and notably, generative AI-assisted content creation. This latter stage was significant for its application of generative AI systems in creating e-books that visually illustrate participants' engagement with everyday consumer technologies. By enabling participants to co-create AI-generated narratives, Stage Two not only documented their lived experiences but also revealed the algorithmic biases embedded in AI-generated media representations. This methodology paper focuses specifically on Stage Two, detailing the participatory, and intersectionality-informed research approaches used in co-creating AI-generated e-books. The subsequent sections outline the theoretical framework, participant, data collection methods, and case study findings. Additionally, ethical considerations and key methodological adaptations made in response to the COVID-19 pandemic are discussed. This ADEEPT project received ethics approval from Western Sydney University (H14057).

## 2 Methodology

This section details the research methods developed, applied and adapted to accommodate participants' needs while remaining flexible in response to evolving research constraints, particularly within the context of the COVID-19 pandemic. While these constraints required adjustments to anticipated methods and timelines, they also created opportunities to refine participatory engagement strategies, allowing the research to be responsive to participants' lived realities (Cargo and Mercer, 2008; Nind, 2020). Furthermore, as the research progressed, the transition from lockdowns to more flexible in-person interactions deepened the understanding of participants' everyday consumer technology use. These iterative adaptations strengthened the participatory nature of the research and allowed data collection methods to remain inclusive, flexible, and aligned with participants' lived experiences. Specifically, the research process itself was reflexively adapted through participant-led refinement of accessibility strategies and engagement practices (Nind et al., 2022). One participant (Nidhi) played a key role in shaping these adaptations through sustained collaboration with the research team.

Building on participatory research principles, the ADDEPT project is also grounded in an intersectionality-informed framework, recognizing that overlapping systems of oppression, such as ableism and linguistic exclusion, shape participants' engagements with everyday consumer technologies, while foregrounding agency by highlighting how individuals resist, adapt to, and negotiate these constraints in everyday life (Crenshaw, 1991; Collins, 2022). Intersectionality and participatory research complement one another by both recognizing CaLD migrants with disabilities as knowledge producers and challenging deficit-based narratives that frame them as passive recipients of technological assistance rather than as capable navigators and users of technology. This methodological approach disrupts such narratives by emphasizing participants' agency in knowledge production and employing multiple modes of engagement, including creative workshops, individual interviews, and AI-generated e-book publications, to center their lived experiences as sites of knowledge production. Drawing on Collins' (Collins, 2019, 2022) conceptualization of lived experience as a source of knowledge, this paper demonstrates how participatory and intersectionality-informed methods can facilitate agency, self-determination, and empowerment in research design. By centering lived experience as expertise, this paper captures how participants navigate, adapt, and redefine accessibility and digital inclusion on their own terms: not as passive subjects, but as active agents shaping their own digital environments.

### 2.1 Participants

Stage Two of the project included 11 CaLD migrants with diverse racial, cultural and linguistic backgrounds, as well as differing migration experiences (e.g., first- or second-generation Australian) which were a subset of those from Stage One. Participants also represented a broad spectrum of disabilities including intellectual, visual, neurological, psychosocial, and physical impairments, reflecting intersections of CaLD migrant identity and disability. Although English was the shared working language, it was not the first language for most participants. Language represented in the project included Vietnamese, Hindi, Arabic, and Cantonese. However, none of the participants requested interpreters for their participation in project activities. Of the 11 participants in Stage Two, this article focuses on five participants whose contributions to the co-creation of AI-generated e-books provide the basis for the case study findings. Detailed profiles of these five participants are presented in the Results section. For clarity, although the participant sample included both CaLD migrants (first-generation) and individuals from CaLD backgrounds (second-generation), we use the term “CaLD migrants with disabilities” throughout this article as a collective descriptor, consistent with the framing of the project.

While all participants are classified as CaLD migrants, it is important to acknowledge that their migration histories and social positions varied significantly which shaped their distinct experiences of digital exclusion and accessibility barriers. For example, migrants with disabilities from racialized backgrounds often face compounded barriers related to racialization, language, and disability in their interactions with everyday consumer technologies and AI-generated representations. In contrast, European migrants from non-English-speaking backgrounds may still experience linguistic and cultural adaptation challenges but are not subject to the same racialized exclusions that non-European CaLD migrants encounter. Furthermore, the category of CaLD migrants in this study also includes individuals born in Australia who actively navigate cultural and linguistic diversity in their daily lives due to familial and community ties. For example, one of the participants, Mani, was born in Australia to parents who migrated from Vietnam. While not a migrant himself, his lived experience is shaped by cultural and linguistic practices that are distinct from those of English-dominant, Anglo-Australian peers. These distinctions are critical for understanding the ways in which structural biases and exclusion are embedded within everyday consumer technologies, AI-generated representations, and accessibility frameworks, all of which operate differently across various social locations. Applying an intersectional lens, the ADDEPT project recognizes that CaLD migrants with disabilities are not a homogenous group, and their experiences with technology, accessibility, and representation are shaped by intersecting social positions including race, language, migration histories, and disability.

Participants were recruited through partner organizations including the Western Sydney Migrant Resources Centre (WSMRC) and YourSide which leveraged their networks to identify potential participants for the ADDEPT project. These organizations approached potential participants via email, providing them with detailed information about the project. Once individuals expressed interest and provided consent, the research team conducted follow-up phone calls to discuss the project further and address any questions. Participants were then sent a participant information sheet in plain English 1 week before participation to ensure they had ample time to review the material and seek clarification.

### 2.2 Data collection

Data collection methods were adapted significantly to accommodate participants' needs, minimize burdens, and address challenges posed by COVID-19 lockdowns, as outlined in the sections below (see also Table 2).

**Table 2:** Summary of methodological adaptations in Stage Two (Phases 3 and 4).

	**Original plan**	**Adaptation**	**Reasons for the adaptation**
Phase 3	Self-documentation	One-on-one interviews, peer discussions, and film/media documentation	Self-documentation was overwhelming and difficult to maintain
	Four-hour workshops	90-min workshops	Zoom fatigue
Phase 4	Independent digital media creation based on self-documented materials	Guided discussions, peer-sharing activities, and interactive facilitation	Needed new ways after self-documentation removal and some found drawing ineffective
	N/A	Addition of post-workshop one-on-one and peer interviews	Creative methods alone were insufficient for some participants

#### 2.2.1 From self-documentation to interactive engagement (phase 3)

Initially designed as a digital self-documentation study, Phase 3 aimed to capture participants' daily technology use across various spaces and activities through personal recordings. To achieve this, Phases 3 incorporated a two-part process: (1) workshops to develop participants' digital self-documentation skills and (2) independent self-documentation where participants recorded their daily technology use through photos, text notes, videos, voice memos, applying the skills learned in the workshops. This approach was essential to align data representation with participants' self-representations, respecting their engagement with technology across diverse digital and physical environments (Trace and Zhang, 2019).

However, many participants found the self-documentation burdensome and stated that recording their daily technology use felt overwhelming and difficult to maintain. Given these challenges, the research team replaced self-documentation with one-on-one interviews and peer discussions, which allowed participants to share their experiences in a more structured and supportive format. To further enhance data collection and provide a comprehensive representation of participants' engagement with everyday consumer technologies, a local disability film and production team was contracted to document participants' technology interactions. These one-on-one interviews, peer discussion and film and media documentation complemented one another and ensured multifaceted depiction of participants' lived experiences. Additionally, this adaptation allowed researchers to gather rich qualitative insights into real-life technology use among CaLD migrants with disabilities, while reducing participant burden by providing alternative ways of expressing technology engagement beyond self-documentation.

COVID-19 restrictions further led to a complete re-design of Phase 3, requiring workshops to move online. For many participants, these online workshops were their first experience with group-based digital learning which demanded continuous adaptation to meet both educational and research objectives (Miller and van Heumen, 2021). Additionally, the prolonged use of virtual platforms contributed to “Zoom fatigue” (Nesher Shoshan and Wehrt, 2022), further exacerbating participant burden. To address these barriers, the research team shortened workshops from 4-h sessions to 90-min sessions. When COVID-19 restrictions lifted in 2022, the research team attempted to return to in-person workshops. However, ongoing public health orders continued to limit large gatherings, which required additional adjustments. First, the venue was shifted from the art gallery to a larger university learning space to comply with health regulations and accommodate social distancing requirements. Second, in-person workshops were restructured into small groups of two to three participants to align with health guidelines and also encouraging more collaborative interaction and enhanced engagement. These adaptations in Phase 3 facilitated a more participant-centered approach by prioritizing accessibility and flexibility to support participant engagement (Cargo and Mercer, 2008; Nind, 2020), while remaining responsive to evolving public health measures.

#### 2.2.2 Evolving digital storytelling and AI-generated narratives (phase 4)

**Figure 1:**
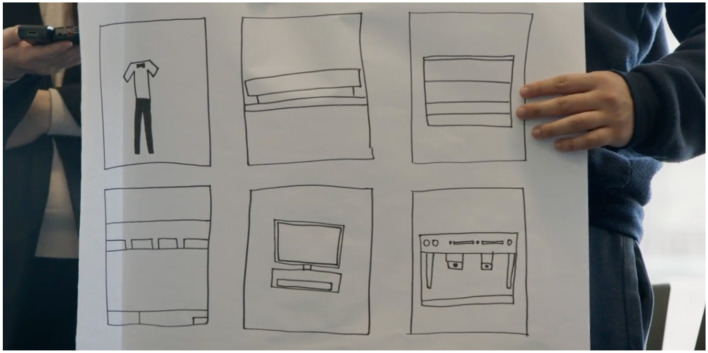
A participant displays their drawings from a workshop. The drawing depicts concisely how they use digital technology in several ways throughout their day-to-day activities.

**Figure 2:**
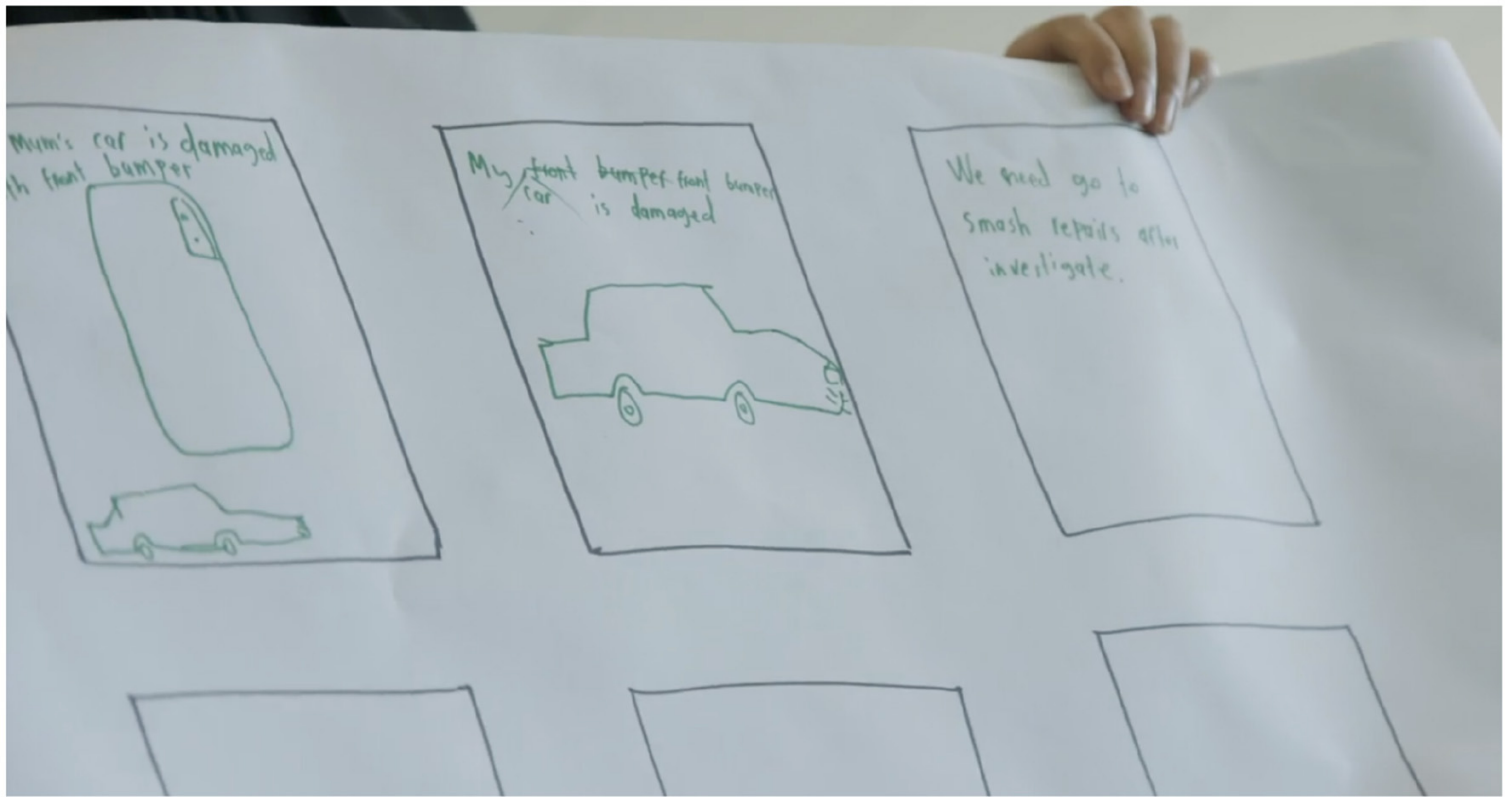
A participant displays their drawings, created during the workshop, to describe their experience with video gaming and its association with their future driving competency.

Phase 4 was originally designed to help participants curate their self-documented materials from Phases 3 and transform them into personal digital galleries through four-hour in-person workshops facilitated by community art professionals specializing in e-book storytelling. However, since the self-documentation component in Phase 3 was removed, the process of Phase 4 was restructured. Without pre-recorded materials to work with, the workshops become more structured and guided to help participants explore alternative ways to express their everyday consumer technology experiences. Instead of independent curation, participants engaged in facilitated discussions and creative exercises with community art professionals providing additional support. Before each workshop, sessions were re-designed to promote participant's artistic and creative expression to explore and distill their daily use of everyday consumer technologies. The use of creative methods was thought to operate as an enabling device to enhance participant communications through drawing and discussion (Kramer-Roy, 2015). However, as workshops progressed, participants responses to creative methods varied significantly. Some participants found drawing and visual storytelling to be effective and engaging and were able to map out their engagement across multiple digital platforms, sites, and applications. For instance, Figure 1 illustrates a participant's ability to visually represent their technology use in a structured and meaningful way. In contrast, other participants struggled to capture their experiences through artistic methods and found drawing cumbersome and ineffective in conveying their personal technology interactions. Figure 2 demonstrates these challenges where a participant attempted to illustrate their journey with video-gaming and how it supported their process of learning to drive. Despite their efforts, the drawing did not fully capture their experience, highlighting the limitations of creative methods for some participants.

Recognizing these challenges, the research team modified workshop content to ensure more inclusive and accessible engagement of all participants. Additional methods including guided discussions, peer-sharing activities, and interactive facilitation techniques were incorporated to better support participants who found artistic methods difficult. Through this iterative process, workshops evolved dynamically and ensured each session was tailored to participants' needs and learning styles.

#### 2.2.2 Introduction of post-workshops interviews

Another key adaptation in Phase 4 was the addition of post-workshop interviews to address participants' difficulties in expressing their experiences solely through creative workshops. These interviews were conducted in two formats: (1) one-on-one interviews prior to workshops to allow participants to reflect on their experiences and share insights into their everyday consumer technology use and (2) one-on-two peer-interviews which enabled participants to engage with others who shared similar lived experiences. While all interviews followed the same two *primary questions, ‘How do you use technology?* and *What problems do you have with using technology'* they allowed interviewees to expand on their responses and provide personalized demonstrations of their technology use and experiences.

A critical finding from the first set of post-workshop interviews was that more than half of participants had limited understanding of AI and its implications for their everyday technology use; the key idea within the ADDEPT project. Since AI was central to Phase 4′s focus on co-creating AI-generated e-books, the research team redesigned the workshop materials to enhance accessibility and comprehension. The revised materials included an Easy English information booklet with visual cues designed to provide a clear and simplified explanation of AI concepts to ensure that participants had a stronger foundational understanding of AI before engaging in AI-generated e-book co-creation activities.

#### 2.2.3 AI-generated e-book co-creation

After the completion of the data collection process across both rounds of workshops (online and in-person) and post-workshop interviews, the research team collaborated with five participants, whose details are provided below in each of the case studies, to co-create multilingual, disability-accessible AI-generated e-books. The co-creation activities were conducted using MidJourney a generative AI program and took place between November 2022 and October 2023 during which MidJourney Versions 4 and 5 were used. As AI tools evolve rapidly, the e-books described in this paper reflect a specific snapshot of generative AI capabilities during that period. Using this platform, these e-books visually and textually represented participants' experiences with everyday consumer technologies. Beyond documenting personal experiences, the co-creation journey provided insights into participants' technological engagement and accessibility needs. First, we learnt about (1) the barriers in navigating digital accessibility and the burden of self-advocacy, (2) the biases embedded in AI-generated imagery, especially in relation to gender, race, disability, age, and body weight, and (3), the role of everyday consumer technologies in fostering agency through self-directed learning, cultural engagement, mobility, and caregiving support.

Contrary to deficit-based narratives that frame CaLD migrants with disabilities as digitally incompetent, participants demonstrated agency, creativity, and resilience in their everyday consumer technology use. Rather relying on external assistance, they actively engaged in peer knowledge-sharing, such as CaLD migrant children teaching their parents how to use new technology, fostering collective empowerment. The following case studies of technology and its multifaceted role in accessible participation, AI prejudice, language skill building, and independent mobility, provide a rich collection of learnings from our co-created research.

## 3 Results

Stage Two of the ADDEPT project was designed to explore how CaLD migrants with disabilities engage with everyday consumer technologies and how AI-generated media representations reflect their lived realities. While intersectionality served as a central theoretical and methodological framework, participants were not explicitly promoted to frame their experience through the lens of multiple intersecting identities such as gender, race, language, disability, age, and body weight. Instead, they were encouraged to narrate their experience in ways that were most relevant and meaningful to them. Each case study highlights a distinct dimension of identity, technology engagement and the challenges participants faced. Table 3 outlines the participants and primary identities that were most explicitly discussed in each case. However, it is important to acknowledge that all cases reflect intersectional lived realities; even when participants foregrounded one identity, their narratives inherently reflected the simultaneous navigation of multiple identities and associated systemic barriers (Bowleg, 2008).

**Table 3:** Participants, primary identities, and key themes in each case study.

**Case Study**	**Highlighted participant(s)**	**Highlighted identities**	**Key themes**
Case One: *Navigating Digital Accessibility*	•Nidhi	•Language •Disability	•Digital accessibility challenges •Multilingual accessibility gaps •Burden of self-advocacy
Case Two: *AI Bias and Misrepresentation*	•Nidhi •Leza •Sadie	•Gender •Race •Age •Disability •Body weight	•AI-generated image misrepresentation •Default aesthetic norms and bias in AI
Case Three: *Everyday Consumer Technology and Agency*	•Mani •Laurance	•Language •Disability	•Assertion of agency through technology •Technology as a tool for self-directed learning, cultural exploration, mobility, and caregiving support

Five participants were involved in the following case studies and co-created AI-generated e-book publications to support accessible knowledge dissemination about their use of technology. These participants and their co-created e-books are introduced below in order in which they appear in the case study findings:

**Nidhi** is a CaLD migrant woman (first-generation) from India with a physical disability and vision impairment in her 20s. She is a disability advocate for a multicultural disability organization in a low socio-economic area of Sydney with significant professional responsibilities in her role and uses technology in both her personal life and career.
**Leza** is a multi-generational white settler Australian woman (second-generation) in her 30s with intellectual disabilities. She lives in a disability-supported residence alongside four housemates and several caregiving staff. She enjoys using technology for activities such as Google, YouTube, email, computer games, as well as for participating in online communities.
**Sadie** is a CaLD migrant gender-neutral person (first-generation) from Lebanon with intellectual disabilities and vision impairment in her 50s. She resided with her mother. She and her extended family had migrated to Australia in her earlier life. She spoke both English and Arabic, with Arabic being the primary language spoken at home. She loved using Facebook and Messenger to connect with and view photos of her family and friends. She sadly passed away before this project was completed, in September 2023. Her friend, Leza, contributed largely to the co-creation of Sadie's story.
**Mani** is an Australia man (second-generation) in his 20s with parents who migrated from Vietnam with intellectual disabilities. He speaks Vietnamese and English. At the time when the workshops were conducted, Mani resided in his family home with his parents and sister. He loves technology, and uses it diversely: exploring interests, making friends, learning a language, and helping his family with different online platforms.
**Laurance** is an Australian man in his 20s (second-generation) with parents who migrated from China living with neurodivergence. He speaks Cantonese and English, with English being his main language, but at home, his parents speak to him in Cantonese. He uses technology to explore his interests, to teach important skills such as driving, and to provide some practical support to his parents.

### 3.1 Case one: navigating digital accessibility

“I think often we hear about sort of making things accessible as though there is just sort of one pathway to doing that but of course, accessibility means very different things for different people. For me, accessibility with vision impairment means translating in Braille, translating audio. It also means translating documents in large print and making the font size larger. It can also mean that you know I used to have things voice recorded as an accessibility format. All things being read out to you by Voiceover on documents and then read out to you as a template email synthetic voice.”

**Nidhi**

Nidhi, who described herself as a CaLD migrant (Indian background) with a physical disability and vision impairment, navigated overlapping systems of exclusion that shaped her engagement with everyday consumer technologies and AI-generative content. As part of the project, Nidhi participated in testing AI-generated e-books (Phase 4) to assess their accessibility for CaLD migrants with disabilities. Her testing revealed significant usability challenges regarding screen reader compatibility and multilingual accessibility. While Nidhi did not experience linguistic barriers herself, as her primary language is English, her insights underscored how accessibility features are often designed with English-dominant users in mind and overlook the diverse needs of multilingual individuals with disabilities.

Nidhi's experiences with everyday consumer technologies provided a foundation for evaluating AI-generated accessibility tools. Having long relied on digital platforms for mobility, communication, leisure and professional advocacy, she had firsthand knowledge of the barriers that individuals with vision impairments face in navigating such technologies. Over the 2-years fieldwork period, Nidhi brought attention to accessibility gaps in mainstream technologies.

“I am vision impaired and use a Samsung phone and an iPad and can use Zoom successfully, but I will need closed captions and all Zoom connection details via dial-in and the link sent to me via email…”

Nidhi's self-disclosure and open sharing of her accessibility needs provided important information for the research team in preparing workshops and co-creating AI-generated e-books. Nidhi provided details not just about her take up of everyday consumer technologies but also, had learnt to give clear instructions of what was required to facilitate this each step of the research. Even though the research team had prepared a set of questions in relation to technology use, capability and challenges in pre-workshop interviews to ascertain accessibility needs of each participant, it was ultimately Nidhi who had to instruct, negotiate, and advocate each step of the process, an experience commonly reported by individuals with disabilities (Konrad, 2021).

Through these discussions, it became clear that the everyday consumer technologies and AI-generated e-books presented overlapping accessibility challenges. For example, while Nidhi was able to read text on digital platforms, she found it very difficult to see images and often relied on voice-over features to navigate her digital devices. Additionally, her screen reader was incompatible with certain platforms, such as Microsoft PowerPoint. She pointed out that she was required to convert PowerPoint presentations to Word or other formats to be compatible with her screen reader software. Nidhi's continued participation in the workshops revealed how non-user-centered accessibility forces individuals with disabilities to be highly adaptive at the expense of ease and efficiency. Her experiences echoed research findings that accessibility accommodations are considered “justifiably excludable”, and are therefore “justifiably absent” from mainstream digital infrastructures (Titchkosky, 2011; Konrad, 2021).

“We never get accessible information in our own accessible format. You go around and ask ‘are you happy with the information in the format? “Usually it is no. Translating resources in different information, to braille, audio and voice recording, voice out, text size accessible content - formal [language] translation, organizations say they don't have time or funding.”

As such, fatigue around communicating access in daily life is a recurring experience for people with disability, because requesting and obtaining appropriate access requires several people with disabilities which have been described by Konrad (2021) as performing “publicly suitable” disability, navigating reactions to disability, negotiating the value of accessibility with others, and the pedagogical responsibility, wherein individuals with disabilities must teach others about accessibility in order to obtain it. There is therefore a significant emotional, mental, and physical burden of access for people with disabilities, which serves as an additional barrier to achieving and using day-to-day technology, and underlies the partially-accessible nature of much digital technology (Konrad, 2021).

What we will see in future cases described in this study, is the exacerbating impact of additional barriers caused by intersecting identities which affect an individual's ability to negotiate accessibility (Konrad, 2021; Mack et al., 2022; Reyes-Cruz et al., 2020). Accessibility requires continuous, clear communication; yet for CaLD migrants with disabilities whose primary language is not English, this process is further complicated by language barriers, placing an even greater burden on them to articulate and negotiate their access needs (Mack et al., 2022; Rink, 2024). Such Anglo-centric technological infrastructures privilege Western, English-speaking users, further widening the digital divide. As a result, CaLD migrants with disabilities often must self-navigate, adapt to, and compensate for inaccessible designs rather than having their needs systematically addressed (Goggin and Soldatić, 2022; Ned et al., 2024).

In summary, Nidhi's experience highlights a fundamental limitation in digital accessibility framework: while many technologies incorporate accessibility features such as screen readers, these tools are often incompatible across different platforms, therefore, users must manually adapt their workflows. Standardized accessibility solutions are deemed to be designed with a one-size-fits-all approach while failing to accommodate the diverse needs of individuals with different types of disabilities. Furthermore, Nidhi's observations underscore the linguistic exclusivity of many accessibility tools, which prioritize English and offer limited support for other languages. This means that users are required to switch platforms or rely on external sources such as Google Translate, to navigate and comprehend digital content. These challenges are particularly pronounced for CaLD migrants with disabilities, especially those with vision impairments who do not primarily speak English.

To address the accessibility barriers in co-creating AI-generated e-books in Phase 4, the research team adopted a user-led adaptation by actively collaborating with Nidhi to develop solutions that suited her needs and preferences. Throughout the research journey, a research assistant maintained ongoing follow-ups with Nidhi to address any access issues and adapt research materials as needed. Proactive strategies, such as phone calls were used to increase the accessibility and transparency of information, while also building rapport. Nidhi's engagement with digital platforms further demonstrates the needs for participant-driven accessibility solutions. During the first workshop, Nidhi openly shared her preference for using Facebook as a documentation tool rather than Padlet, which had been provided by the research team. Although Padlet met all Nidhi's accessibility requirements, her preference highlighted a gap in accessibility design: even when a tool is technically accessible, its unfamiliarity can create an additional barrier. Through ongoing, proactive communication, the research team adjusted documentation methods based on Nidhi's feedback and adopted Facebook as the main documentation tool instead of Padlet to better align with her existing digital practices. It should be noted that while these strategies were adapted by the team to ensure a smooth and adapted process to best suit Nidhi's needs, this ease of communication and enhanced efforts toward accessibility are often absent in everyday consumer technologies, and even in research and academic contexts (Isaacson, 2021; Konrad, 2021). This underscores the need for accessibility framework that go beyond compliance and instead prioritize user-driven, contextually adaptive solutions that integrate accessibility meaningfully integrated into technological design and research practice.

In conclusion, Nidhi's experiences navigating digital accessibility revealed gaps in mainstream accessibility frameworks. While many platforms claimed to be “accessible”, her engagement with everyday consumer technologies and AI-generated e-books exposed persistent barriers in screen reader compatibility and multilingual access. Even when accessibility features are available, they are often inconsistent, difficult to navigate, and linguistically exclusive. Ultimately, Case One provided critical insights into digital accessibility which set the stage for Case Two which expands on these discussions by examining of AI-generative images and explores the biases embedded in AI-generated visuals showing how generative AI perpetuates systemic biases in gender, race, disability, age, and body weight representation.

### 3.2 Case two: AI bias and misrepresentation

Despite advancements in AI and machine learning, generative AI systems continue to reproduce and amplify societal biases, especially when representing individuals who experience multiple, intersecting forms of exclusions. This case study explores how AI-generated misrepresentation of gender, race, disability, age, and body weight shaped the digital portrayals of Nidhi, Leza, and Sadie, and highlights the systemic limitations of AI in representing diverse identities without perpetuating existing social hierarchies.

#### 3.2.1 Journey of co-creation with Nidhi: “autonomy, freedom, and mobility”

During the co-creation of an experience-based AI-generated e-book, Nidhi, a visually impaired disability advocate of Indian background, faced several challenges while creating AI-generated images that accurately represented her intersecting identities (Shekaran et al., 2023).

**Figure 3:**
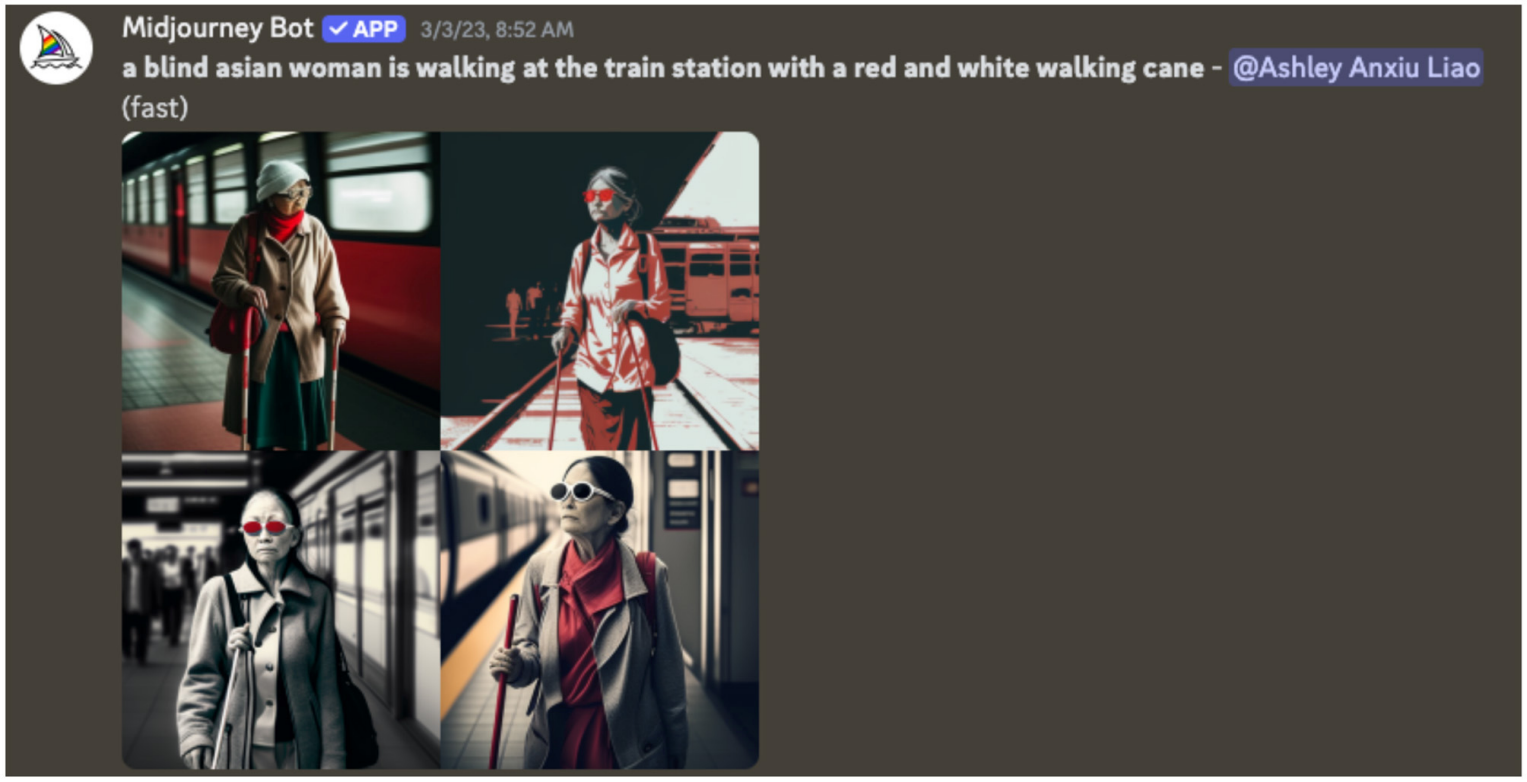
A screenshot of one of the preliminary visual image outputs from generative AI. The images display an elderly East Asian woman walking with a cane and dark glasses.

**Figure 4:**
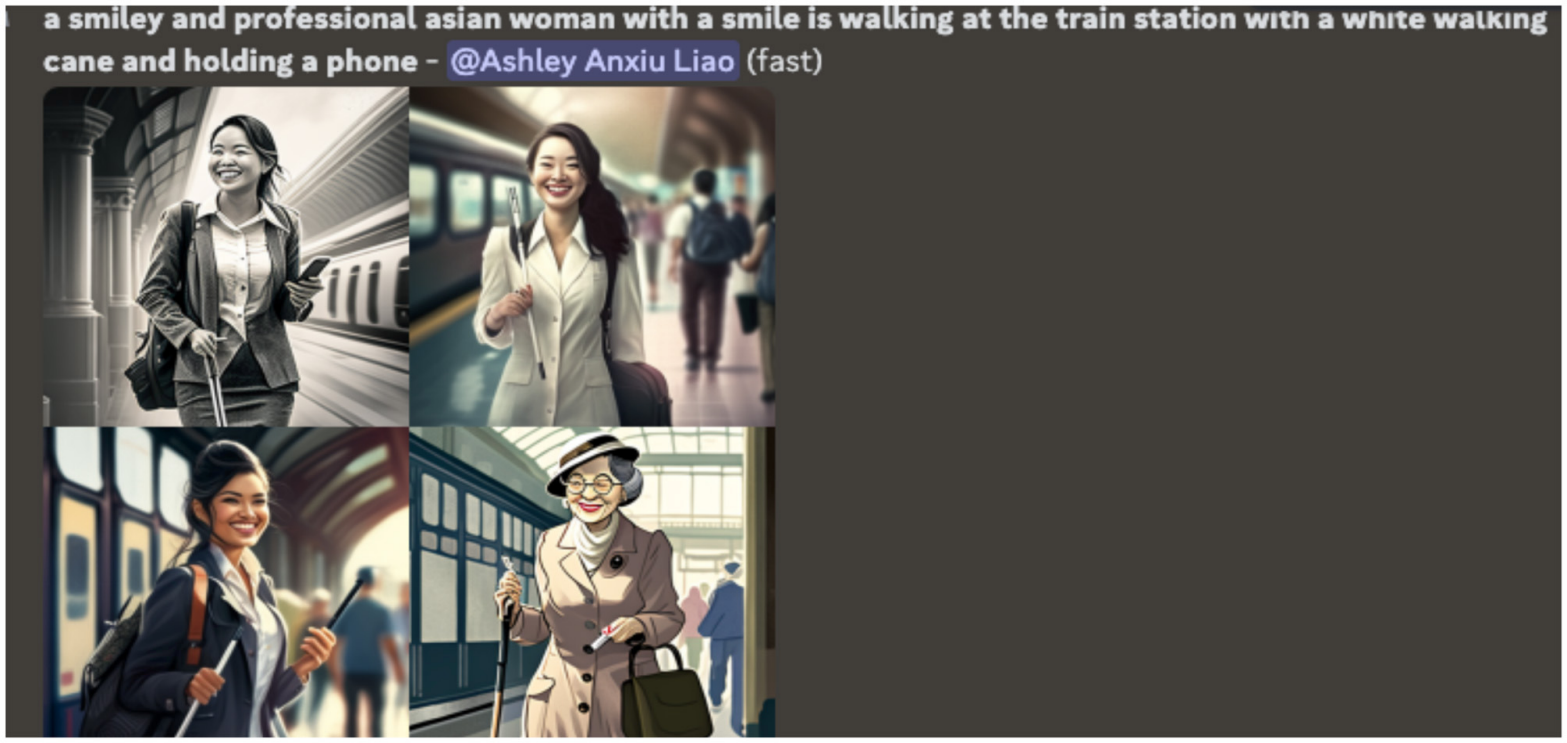
A screenshot of another attempt at generating a visual representation of Nidhi. The images display only East Asian women.

**Figure 5:**
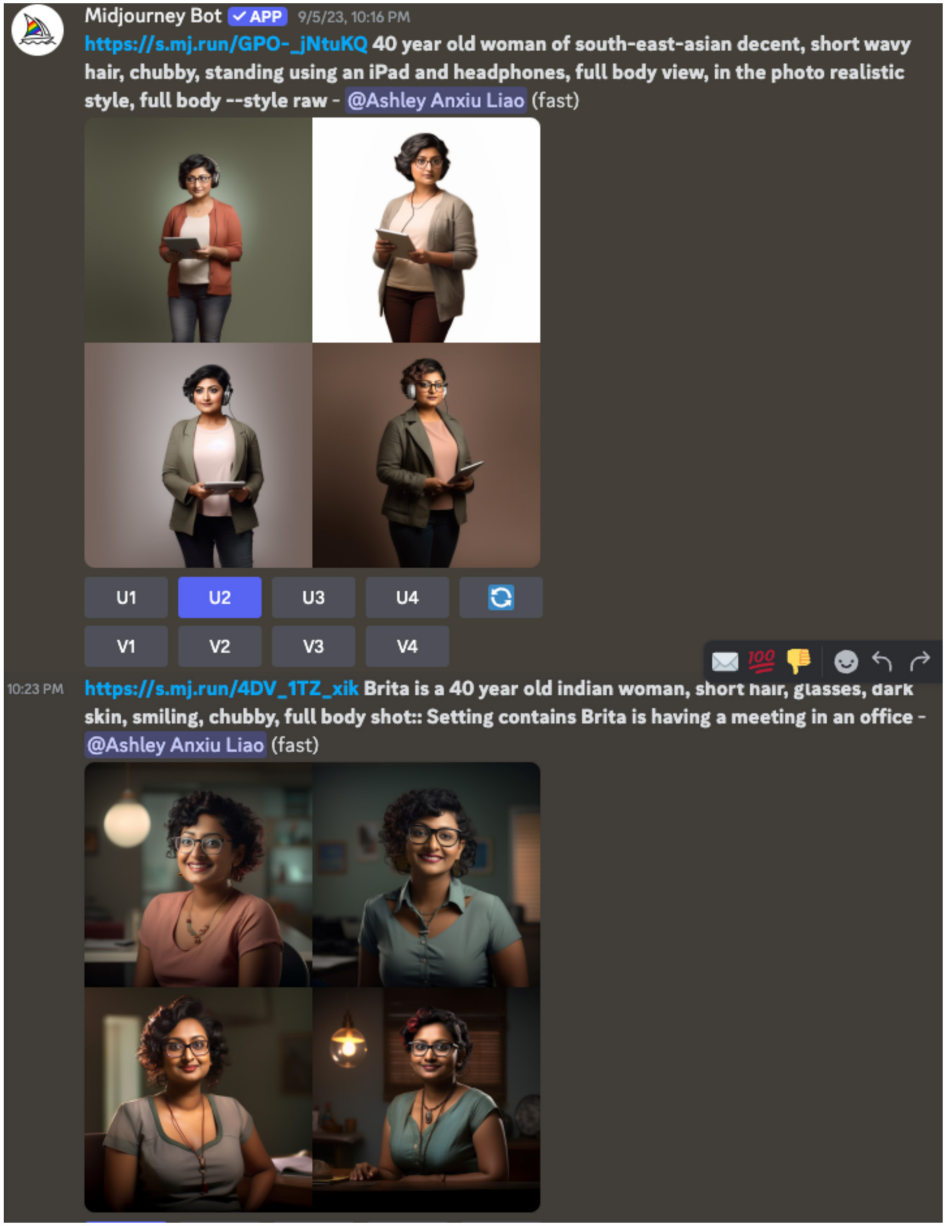
The final iterations of AI generative visualizations of Nidhi, using detailed, explicit descriptors.

Figures 3–5, the AI visualizations created for Nidhi, reveal that the AI system interpreted descriptive terms including “woman”, “Asian”, and “blind” through a lens of preconceived biases. Specifically, the Figure 3 demonstrates the AI's automatic association between gender, race, disability, and aging and depicts Nidhi as an elderly woman, despite no age-related descriptor being included in the prompt. This suggests that AI visualization tools reproduce dominant societal narratives that equate the intersection of Asian women with disabilities with older age, rather than recognizing disability as a lifelong or otherwise acquired earlier in life.

To address these inaccuracies, the research team refined the input descriptors by adding terms such as “professional” and “smiley” to better reflect Nidhi's self-identification. However, this introduced another layer of bias (see Figure 4): the AI began generating images of individuals presumed to be of East and Central Asian descent, indicating long-held stereotypes around the category of peoples from this geographical location (East and Central Asia), where East and Central Asian people are considered “more Asian” and that South Asian people are less Asian, or not considered Asian at all. Public rhetoric calls out the targeted position of South Asian communities, such that race and colorism impacts their experience of Asian identity in AI-generated representations. Within these systems, South Asian identities are frequently marginalized or rendered invisible, as dominant data sources and algorithmic training sets privilege lighter-skinned East and Central Asian phenotypes as the default “Asian” representation. This reproduces and reinforces existing racial hierarchies within the category of “Asian”, compelling users to engage in additional labor to assert the visibility and accuracy of their identities.

The most accurate representation (Figure 5) was only achieved after explicitly detailing every aspect of Nidhi's identity including her disability, profession, and self-perceived characteristics, demonstrating that to authentically represent diverse identities, it requires the users to over-specify attributes to counteract the pre-existing biases within the algorithm. Ultimately, the burden falls on users to “correct” intrinsic biases to generate accurate depictions of their intersecting identities.

#### 3.2.2 Journey of co-creation with Leza: lifelong learning and remembering Sadie Daher

Like Nidhi, Leza and Sadie encountered significant challenges in generating AI images that accurately reflected their intersecting identities. This section explores how AI's default aesthetic norms and embedded biases influenced their digital representations, requiring extensive prompting and user intervention to achieve more accurate portrayals (Grundy et al., 2023).

##### 3.2.2.1 About Leza

Leza resided in a disability-supported accommodation for persons with intellectual disabilities with four housemates and had minimal contact with family members. She identified her cultural background as Australian, specifically as multi-generational white settler Australian. Her engagement with everyday consumer technology was shaped by both personal interest and structural constraints with her living environment. Leza experiences with technology were mixed. She owned a computer and a smartphone, which she used for leisure and entertainment, such as watching YouTube videos and listening to music. She appreciated YouTube's personalized recommendations and keyword search functions which allowed her to curate content based on her interests. However, her technology use was closely monitored by house staffs, especially regarding social media, email access, and online communication platforms. While this is a commonly encountered form of online risk mitigation and safety measures taken by caregivers of individuals with developmental or intellectual disabilities, they contributed to feeling of restriction, digital exclusion, and a lack of autonomy (Chadwick, 2022).

##### 3.2.2.2 Co-creation of stories with Leza

**Figure 6:**
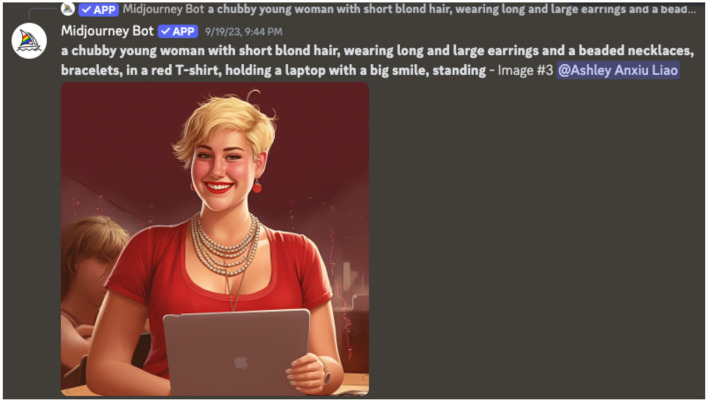
A screenshot of the final visual output agreed upon by collaborators to represent Liza, after significant difficulties in AI generation of a representative image of Leza's “true” self.

Two years after the initial workshops, Author Ashley Liao visited Leza at her shared home to co-create her AI-generated e-book. As part of this co-creation process, photographs of Leza, her computer, and her screen were taken to inform the development of AI generated images. However, the AI-generated images had the tendency of making the image of her character look like a ‘Barbie doll', which did not reflect her identity nor her preference. This Barbie doll-like depiction exemplifies stereotypical portrayals of femininity, projecting Eurocentric body ideals shaped by the intersection of whiteness and sexism, which have been critiqued for perpetuating narrow, hyper-feminized and exclusionary standards of women (Sutko, 2020). To counteract this bias, the research team continuously refined the AI prompts with Leza's ongoing feedback. The final prompt was structured as: “a chubby young woman with short blond hair, wearing long and large earrings and a beaded necklace, bracelets, in a red T-shirt, holding a laptop with a big smile, standing” (Figure 6). Despite these refinements, the AI-generated images continued to misrepresent Leza' self-image. To ensure that readers saw an authentic representative image of Leza, she ultimately chose to use her personal photograph for the book cover, while incorporating AI-generated images throughout the internal pages.

Leza's experience highlights the persistence of AI biases that conflate womanhood with hyper-feminization, thinness, and Eurocentric beauty ideals. It also demonstrates the labor required by users to repeatedly intervene and correct algorithmic misrepresentations.

#### 3.2.3 Remembering Sadie: technology, family, and representation

##### 3.2.3.1 About Sadie

Sadie lived with her mother and came from a Lebanon-Australian background, having migrated to Australia with her extended family during childhood. She is bilingual, speaking both English and Arabic, with Arabic being the primary language spoken at home. While her siblings had since moved out to start their own families, Sadie remained connected to them through everyday consumer technologies. Sadie particularly valued Facebook Messenger because she could access the photos of her nieces and nephews and participate in family conversations using emojis. Her digital interactions with her family were often conducted in both English and Arabic, demonstrating the linguistic and cultural integration within her family network. This aspect of family interaction via digital technology was important to Sadie, as it enabled her to connect with her family while living apart from one another. Despite living with an intellectual disability and restricted vision, Sadie did not identify these impairments as barriers to the use and enjoyment of everyday consumer technology. When asked if she found anything difficult about technology, Sadie always responded that she was unsure which suggest that her lived experiences of living at the intersection of South-east Asian gender-neutral person with disabilities did not hinder their digital participation in the ways often assumed by accessibility discourses.

During the project, as the team reached out to collaborate on her AI-generated e-book, Sadie was hospitalized with a severe illness. Her siblings communicated on her behalf, shared her excitement about the storybook and consented for her name to be included. However, before the team could visit her with a draft, Sadie sadly passed away. The completed e-book was launched with her immediate family in attendance, including her mother, siblings, cousins, and extended relatives.

##### 3.2.3.2 Co-creation of Sadie's stories with Leza

**Figure 7:**
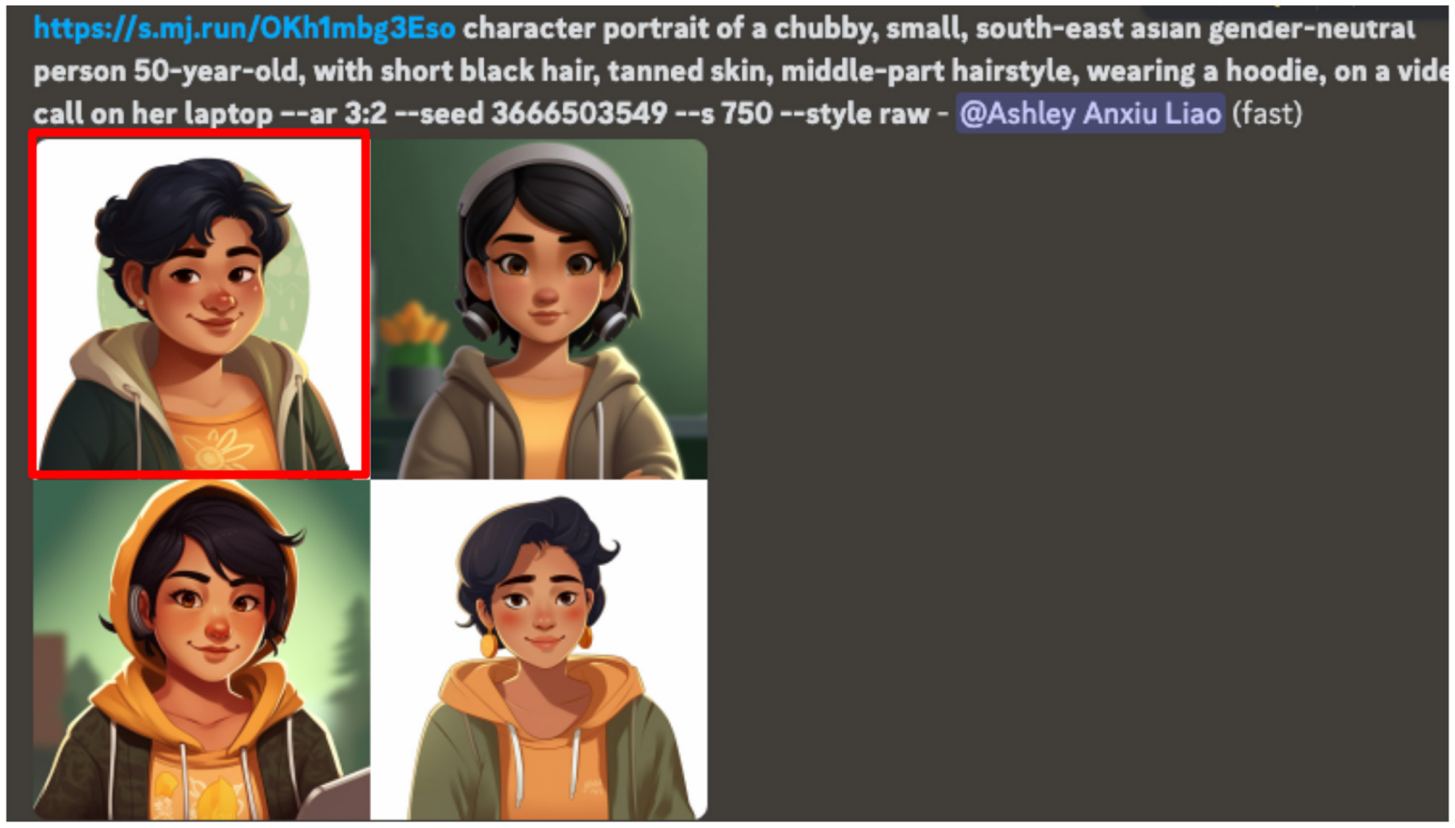
The final prompts used to create the AI image of Sadie, with the selected image for the books highlighted in red.

Following Sadie's passing, her close friend Leza played an integral role in contributing to Sadie's story by recalling memories of their shared experiences with technology and actively selecting AI-generated images to represent Sadie. A series of AI-generated portraits were produced using the prompt: “a chubby, small, south-east Asian gender-neutral person, 50-year-old, with short black hair, tanned skin, middle-part hairstyle, wearing a hoodie, on a video call on her laptop”. These prompts were deliberately crafted to counteract potential AI biases in gender, race, and body representation that had been observed in Nidhi's and Leza's cases (Figure 7). Furthermore, the term “gender-neutral” was intentionally included to mitigate the risk of gendered bias in AI-generated representation particularly in hairstyle and clothing (Figure 7). However, despite specifying Sadie's age as 50 years old, the AI-generated images depicted them as significantly younger. This contrasts with Nidhi's case (see Figure 3) where AI automatically associated vision impairment with older age and portrays Nidhi as elderly despite no age-related descriptor being included. These patterns suggest that AI-generated visualizations rely on ingrained societal assumptions: associating vision disability with old age in Nidhi's case while failing to recognize middle-aged individuals like Sadie unless they exhibit stereotypical aging features such as wrinkles or gray hair. After extensive prompts and discussion with Leza, the highlighted image in Figure 7 was selected as the most appropriate representation of Sadie which aligned with Leza's perspective and Sadie's family's wishes.

Ultimately, Sadie's case highlights the emotional, ethical, and representational complexities of AI-generated imagery in the context of digital memorialization. Furthermore, it raises questions about who gets to be represented, how identities are visually reconstructed, and what it means to create a digital legacy for those no longer present to shape it themselves.

Taken together, the AI-generated e-book co-creation process with Nidhi, Leza, and Sadie shows the complexities of AI-generated imagery, and the way algorithmic biases shape digital representation. While AI holds the potential for personalized and inclusive visual storytelling, Case Two shows how current generative models often perpetuate pre-existing societal stereotypes across multiple social identities including gender, race, disability, age, and body weight. Furthermore, it highlights the need for AI-generated imagery to prioritize diverse intersecting identities from the outset, rather than relying on users to correct systemic biases through repeated iterations, an issue criticized widely in discussion of how structurally disadvantaged individuals are often expected to advocate for their own inclusion (Konrad, 2021).

### 3.3 Case three: everyday consumer technology and agency

Everyday consumer technologies play a critical role in fostering self-directed learning, social engagement, and independence for CaLD migrants with disabilities. This case explores how Mani and Laurance, two participants with distinct technological engagement, used everyday consumer technology to navigate language learning, cultural connections, and mobility. Their experiences challenge dominant narratives that often depict CaLD migrants with disabilities as passive technology users, instead demonstrating their active participation in digital and physical space through such technologies, not only for personal development and as support providers for their families.

#### 3.3.1 Journey of co-creation with Mani: “languages, learning, and inclusion”

The following case examines the lessons learned from co-creating “Languages, learning and inclusion” alongside Mani (Chung et al., 2023). Mani's story, as represented in his AI-generated e-book, highlights the role of technology in language acquisition and cultural engagement through AI-driven translation tools (e.g., Google Translation) and language learning application, Duolingo. His self-directed learning of Japanese and his role in assisting his mother in learning English demonstrate the reciprocal nature of digital literacy, where individuals with disabilities not only benefit but also facilitate technological engagement for those around them.

##### 3.3.1.1 About Mani

Mani, who lives with an intellectual disability, was born in Australia with parents who migrated from Vietnam. He is bilingual in Vietnamese and English and is self-taught in Japanese. At the time when the workshops were conducted, Mani resided in his family home with his parents and sister. When COVID-19 restrictions eased, Mani's elder sister drove Mani and his mother to attend the in-person workshops held in the second year of the project. Mani had an active social life and attended various group programs designed for people with intellectual disabilities throughout the week, including a weekly computer-interest group. He also engaged with everyday consumer technology to learn language and to use social media networking. Mani shared his interest in Japanese culture and animation which influenced his self-directed efforts to learn Japanese through platforms such as Google Translate and Duolingo. In addition, Mani helped his mother, a native Vietnamese-speaker, learn English using such technology.

##### 3.3.1.2 Co-creation of Mani's story

**Figure 8:**
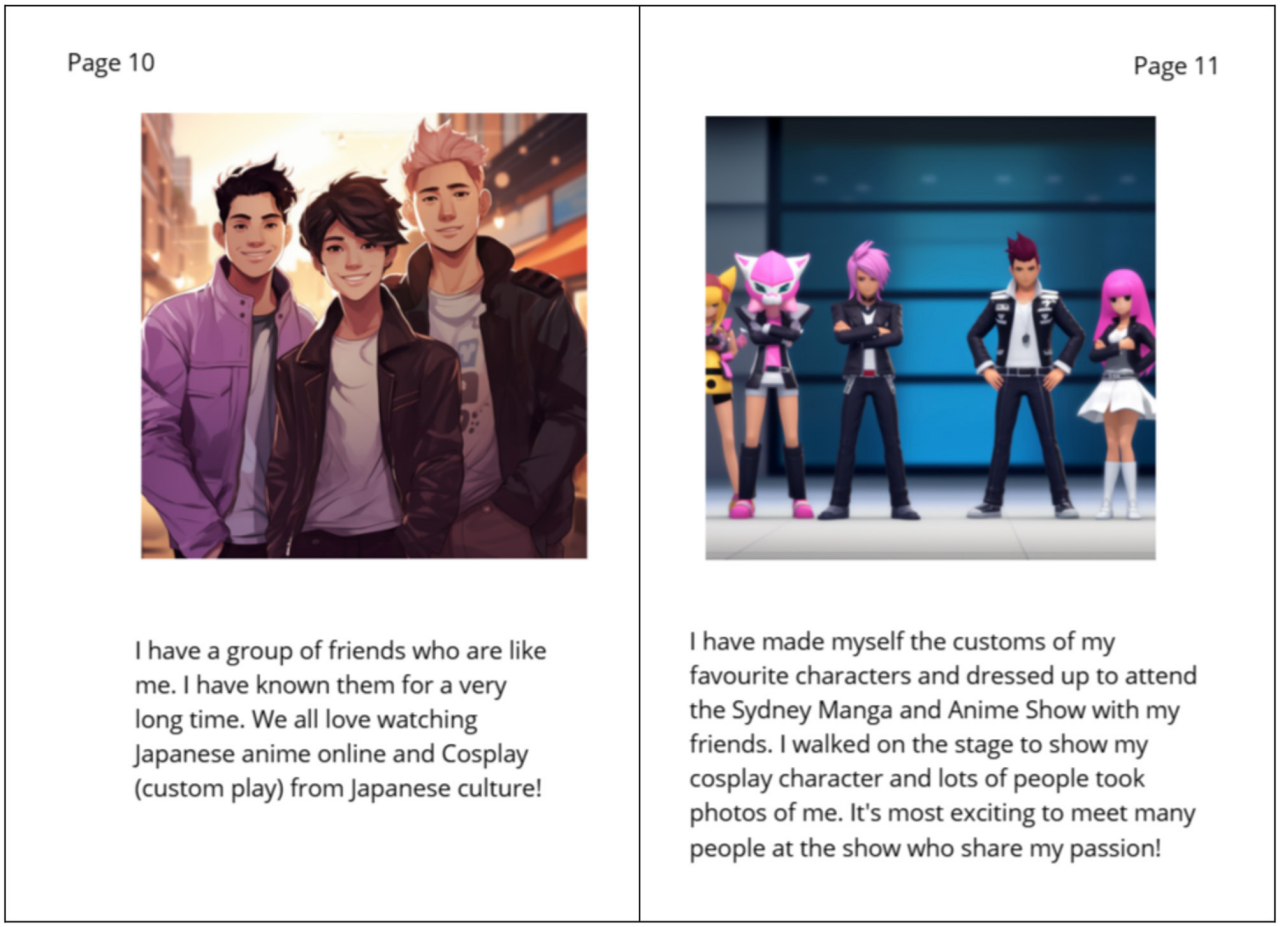
“Languages, learning and inclusion” (Chung et al., 2023). Mani describes his interests and his social community around anime and cosplay.

**Figure 9:**
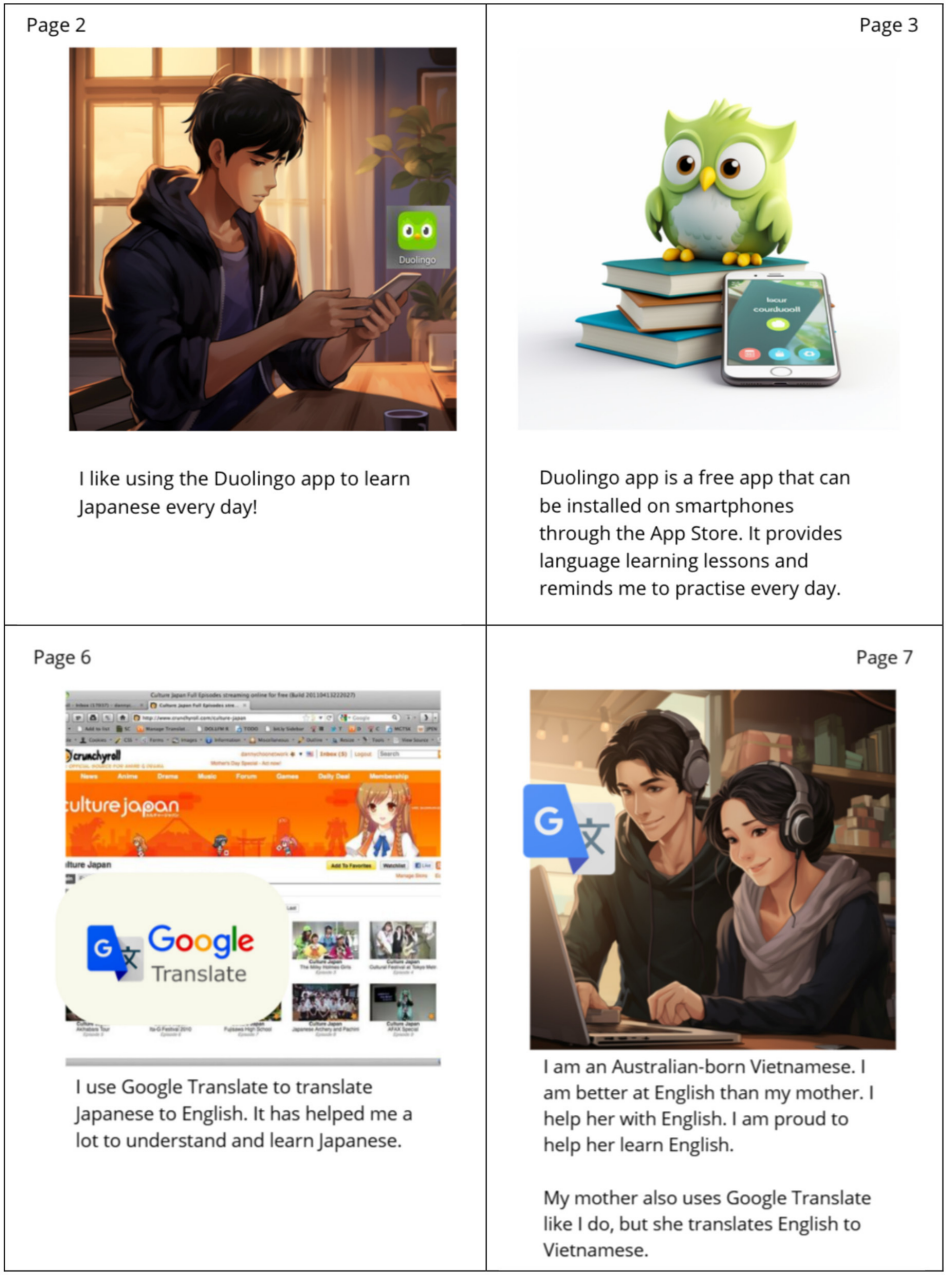
“Languages, learning and inclusion” (Chung et al., 2023). Demonstration of Mani's use of Duolingo and Google Translate for language learning and teaching.

As part of the AI-generated e-book co-creation process, Mani's experiences with language learning and cultural engagement were central themes. During the workshops, Mani highlighted his recent achievement of creating and wearing costumes of his favorite video game characters (also known as “cosplay”) which he presented on stage along with his friends at the Sydney Manga and Anime Show (Figure 8). This was included as an integral part of his story around everyday consumer technology, especially because of the strong involvement of the technology in developing these friendships and contributing to their shared interests in anime and video games. It also served to inspire and drive his creative costume in cosplay. Beyond cosplay, Mani's technological engagement was driven by his passion for Japanese culture and language. During post-workshop interviews via Zoom, he described his commitment to daily language learning using Duolingo and highlighted how the app's reminders helped him stay consistent in his practice every day (Figure 9) (Chung et al., 2023). His aspiration to visit Japan 1 day further strengthened his motivation to learn the language (Figure 9).

In addition to his own language learning, Mani played a unique role as a digital mediator for his family. He helped his mother, a native Vietnamese-speaker, in learning English. Through Duolingo, Mani guided her in navigating the app, answering educational prompts, and making sense of new vocabulary (Figure 9). Mani was very proud of his contribution to his mother's language development. He was also able to provide similar technological supports for many of his family members, helping them to navigate through unfamiliar software and platforms, including government websites, public transport apps, language learning apps, social media, Google Maps and their favorite mobile or digital games.

#### 3.3.2 Journey of co-creation with Laurance: gaming, driving, and independence

Independent mobility in the context of disability was a strongly represented theme throughout this project. This section examined the stories of Laurance, whose lived experience with disability includes roles as both a care receiver, and a care giver, facilitated through digital technology (Trieu et al., 2023). As a bilingual individual, Laurance's relationship with technology extended beyond personal use. He played an active role in supporting his aging parents, challenging conventional assumptions of dependence. His story illustrates how individuals with disabilities can serve as key facilitators in their families' digital and mobility experiences.

##### 3.3.2.1 About Laurance

Laurance is an enthusiastic and autonomous adult who lives with neurodivergence. Laurance has a keen interest in trains, cars and gaming. At the time he participated in the workshops, he was living with his family and preparing to enter the workforce. Alongside the research team, Laurance co-created the “Gaming, driving and independence” e-book (Trieu et al., 2023), which explored his evolving relationship with technology, mobility, and caregiving roles.

##### 3.3.2.2 Communication

Laurance is bilingual in English and Chinese Cantonese. English is Laurance's main language, which he uses to receive auditory and written information. Laurance can express himself by speaking and writing in English. Laurance's expressive oral communication appeared at times to be at a basic level, when he spoke using short sentences, phrases or single words. Laurance's parents speak to him in Cantonese at home which he can understand, such as following instructions. At times, Laurance provides practical support to his parents. Often, individuals with disabilities are depicted as being hyper-dependent on caregivers, often ignoring many of the important and supportive roles that these individuals have in supporting those around them (Flynn, 2021). In many migrant communities, first-generation migrant parents will rely on their children for support in communicating with others in their new home; children act as language brokers and sociocultural mediators for their parents (Bauer, 2016; Orellana, 2009). Laurance and many children of migrants thus adopt a partial caring role at an age and in a way that most children of non-migrants may not experience (Orellana, 2009); this role persists even for a person experiencing intellectual or developmental disability.

##### 3.3.2.3 Co-creation of Laurance's story

Throughout the workshops, Laurance demonstrated his intense interest in cars, trains and gaming. Not only were these interests frequently addressed during Laurance's participation of group activities and individual interviews, but Laurance was also observed to spend a significant amount of time during the workshops immersing himself in these interests via his iPad and phone. This included activities such as browsing car manufacturer information, looking up train timetables, and playing mobile games, using web browsers, apps, and a range of other media platforms. In the first workshop, Laurance demonstrated his technology usage through a drawing of himself using his phone to take photos of a car accident in which his mother was the driver, and he was a passenger and witness. Laurance later drew a conceptual design of a car, equipped with an autonomous driving function, with the aim of this function preventing accidents like the one he experienced.

**Figure 10:**
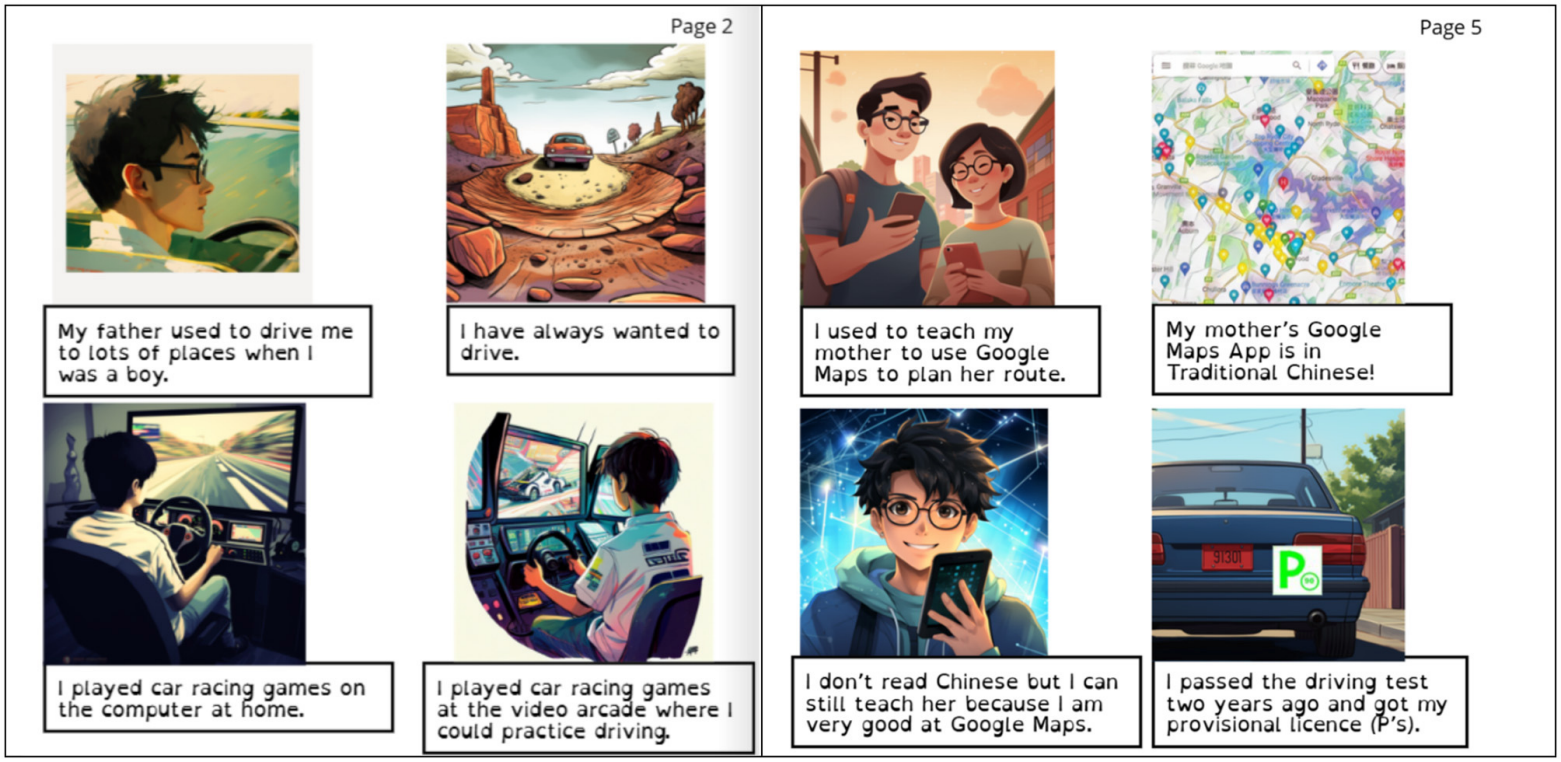
“Gaming, driving and independence” (Trieu et al., 2023). Laurance's use of technology to learn to drive, find directions, and support his parents.

Laurance's gaming interests also played a pivotal role in developing his real-world mobility skills. From an early age, his parents introduced him to a virtual reality driving game with a steering wheel controller, which he used extensively (Figure 10). Over time, this setup provided an accessible and engaging platform for him to develop the necessary skills and confidence in driving. By the time of his e-book's creation, Laurance had successfully passed his driving test and had adopted the role of the primary driver for his aging parents (Figure 10). This marked a significant milestone in his independence and challenged traditional narratives that position individuals with disabilities solely as care recipients. Instead, his story highlights the transformative role of everyday consumer technology in fostering mobility, independence, and the redistribution of caregiving responsibilities within families (Soldatic et al., under review).^1^

Taken together, the experiences of Mani and Laurance illustrate the way in which migrants with disabilities actively shape their engagement with everyday consumer technologies, challenging dominant narratives that portray them as passive users. Their stories highlight the critical role of digital agency where individuals not only adapt to technology but also leverage it to assert independence, navigate language barriers, and facilitate caregiving roles. Furthermore, their narratives reinforce the need for an intersectionality-informed, agency-based approach in understanding technology use and recognize that accessibility is not simply about providing technological tools but ensuring that these tools reflect the diverse realities and needs of their users. Through self-directed learning, and the redistribution of caregiving responsibilities, Mani and Laurance exemplify how CaLD migrants with disabilities are active agents in its use, transformation, and integration into daily life.

## 4 Discussion

Based on the lessons learned through the participatory and intersectionality-informed ADDEPT project, we propose several methodological parameters to guide future disability-inclusive, participatory research. While the parameters focus primarily on participatory practice, we encourage researchers to apply them within an intersectionality-informed framework that explicitly attends to how ableism, racism, xenophobia, linguicism, and other systemic barriers shape participants' experiences and engagements.

First, it is important to maintain a careful balance between in-depth participatory engagement and participant burden. While sustained engagement can enrich the research, researchers must continuously monitor and mitigate potential cognitive, creative, and emotional demand on participants. This is particularly important when working with participants who face multiple systemic barriers which may compound the labor required for research participation. In this project, Nidhi's experience demonstrated how individuals with disabilities are often required to advocate for their own accessibility at every stage, even within research that aims to center their voices. Furthermore, as she described, the English-dominant design of many technologies compounds these barriers for CaLd migrants with disabilities, contributing further cognitive and emotional demands.

Second, methodological flexibility is essential to support inclusive and sustained participation. Research designs, timelines, and engagement methods should be adapted responsively to participants' need and contextual constraint. In the ADDEPT project, the shift to online workshops due to COVID-19 required significant restructuring of engagement strategies. Reducing workshop duration, providing alternative modes of participation and maintaining individualized follow-ups were critical in addressing these barriers, ensuring accessibility and sustained involvement in the project.

Third, sustained engagement and trust-building are critical. Developing long-term relationships with participants fosters deeper, more meaningful research outcomes and promotes participant agency and voice particularly for those who experience intersecting marginalization that may have fostered historical distrust toward researchers and institutions. In the ADDEPT project, sustained engagement and trust-building enabled Nidhi's collaboration over 2 years which helped refine research accessibility strategies and supported Leza's involvement in shaping an authentic representation of Sadie's story in co-created outputs.

Fourth, participant-led identification of accessibility needs and systemic biases should be prioritized. Engaging participants as experts in these areas supports the co-creation of more inclusive knowledge and ensures that research processes and outputs are grounded in lived experiences. In this project, Nidhi, Leza, and Sadie played an active role in identifying and correcting AI-generated image biases, as well as refining prompts to achieve more accurate representation. However, this process placed an undue burden on individuals who were already navigating systemic barriers, underscoring the need to carefully balance participatory engagement with participant burden as discussed in the first parameter.

Fifth, researchers must remain aware of the persistent limitations of accessibility tools. It is important to develop participant-informed, contextually adaptive approaches that address the intersectional needs of diverse users. For example, while assistive features such as screen readers are widely available, their incompatibility across platforms forced participants such as Nidhi to engage in additional adaptation efforts. Furthermore, the English-first design of many accessibility features excluded participants whose primary language was not English, increasing the burden of navigating exclusionary technological design.

Finally, participatory research should center participants' agency and expertise. Moving beyond deficit-based framings, researchers should recognize the value the active roles participants can play in shaping digital environments, advocating for accessibility, and building community capacity. In this project, participants demonstrated significant agency and expertise; Nidhi as an active speaker on disability access and as a significant informant for the study; Leza by supporting researchers in developing Sadie's story and giving her a voice; Mani by supporting his parents in learning English and teaching them how to use digital technology to do so; and Laurance by providing practical support and mobility assistance to his aging parents.

Taken together, these lessons emphasize the importance of adopting flexible, participant-centered approaches that account for diverse accessibility needs and minimize participant burden. Furthermore, they highlight that long-term, co-created engagement fosters richer insights and more inclusive research processes and outcomes. The day-to-day use of digital technology for CaLD disability communities is diverse, with added barriers and benefits which may not be otherwise experienced outside of this lens. In conducting participatory, co-creation research with participants with disabilities and CaLD identities, digital technologies served not only as a main topic of inquiry and discussion, but also as tools to engage participants, collect data, and synthesize materials for accessible knowledge dissemination. The methods denoted in this paper contribute to the body of embodied participatory and intersectionality-informed approaches to research and highlight the key understandings of technology use in this context.

## Data Availability

The datasets presented in this article are not readily available because they are for project use only.

## Footnotes

^1^Soldatic, K., Lee, M., Coe, G., and Magee, L. (under review). Agential technologies or agential subjects: Culturally and linguistically diverse (CaLD) migrants with disabilities and their adaptation of everyday consumer technologies. *J. Ethnic Migr. Stud*.
